# A New Nano Adjuvant of PF3 Used for an Enhanced Hepatitis B Vaccine

**DOI:** 10.3389/fbioe.2022.903424

**Published:** 2022-05-10

**Authors:** Pu Shan, Zhibiao Wang, Jilai Li, Duoqian Wei, Zhuan Zhang, Shaojie Hao, Yibo Hou, Yunyang Wang, Shuxiang Li, Xudong Wang, Jing Xu

**Affiliations:** ^1^ National Vaccine and Serum Institute (NVSI), China National Biotech Group (CNBG), Beijing, China; ^2^ Beijing Institute of Biological Products Co., Beijing, China

**Keywords:** PF3, nano adjuvant, ginsenoside Rg1, aluminum adjuvant, hepatitis B surface antigen

## Abstract

Recombinant protein vaccines, with highly pure ingredients and good safety, are gradually replacing some attenuated and inactivated vaccines in clinical practice. However, since their low immunogenicity of the recombinant proteins, adjuvants are often needed to enhance immune response after vaccination. Aluminum adjuvant has been widely used in some vaccines for decades, it can induce strong humoral immunity, but the deficiency of cellular immunity limits its application for some vaccines. Therefore, it is urgently needed to develop novel adjuvant to increase not only humoral but also cellular immune response. To address this, we designed and prepared a new nano adjuvant (PF3) through microfluidization by the combination of saponin (Ginsenoside Rg1) and oil-in-water nano emulsion (NE) in the present study. As compared to aluminum adjuvant, PF3 had stronger humoral and cellular immune induction effect because of high cellular uptake and activization of immune response pathways. Furthermore, PF3 showed better immune enhancement and acceptable biosafety equivalent to that of aluminum adjuvant. In addition, no obvious changes of PF3 were observed in size and zeta potential after 12 weeks storage at 4 and 37°C, demonstrating its high stability *in vitro*. This study provided an adjuvant platform to replace traditional aluminum adjuvant in design of recombinant vaccines.

## Introduction

The importance of adjuvants to vaccines such as recombinant proteins is self-evident. In clinical settings, adjuvants are often incorporated within vaccine formulations through physical or chemical association with antigens (Lee et al., 2020). Aluminum has been on the market for nearly 90 years as a vaccine adjuvant, however, it tends to attach on the membrane rather than entering the dendritic cells (DCs), leading to the absence of intracellular transfer and process of the antigens, and thus limits T-cell-mediated immunity (Peng et al., 2020). The development trend of adjuvants clearly shows that single components, such as aluminium adjuvants, cytokine or chemokine adjuvants and TLR (Toll-like receptor) agonists, often fail to meet the needs of various infectious diseases. Thus, combined adjuvants show great promise in the development of adjuvants in the future. For example, AS01_B_ is one type of combined adjuvant that is composed of a liposome, MPL and QS21 and AS01_B_ has been employed in a recombinant herpes zoster vaccine, Shingrix. It reduced the risk of herpes zoster infection by 97.2% in an elderly population aged 50 years and older and by 96.6–97.9% for all age groups (Syed, 2018). However, the efficacy of the marketed live attenuated herpes zoster vaccine Zostavax was only 69.8% in the population aged 50–59 years, and it was even lower in the elderly population aged over 60 years (James et al., 2018). Therefore, combined adjuvants showed the tremendous potential in boosting immune response of vaccines.

**FIGURE 1:**
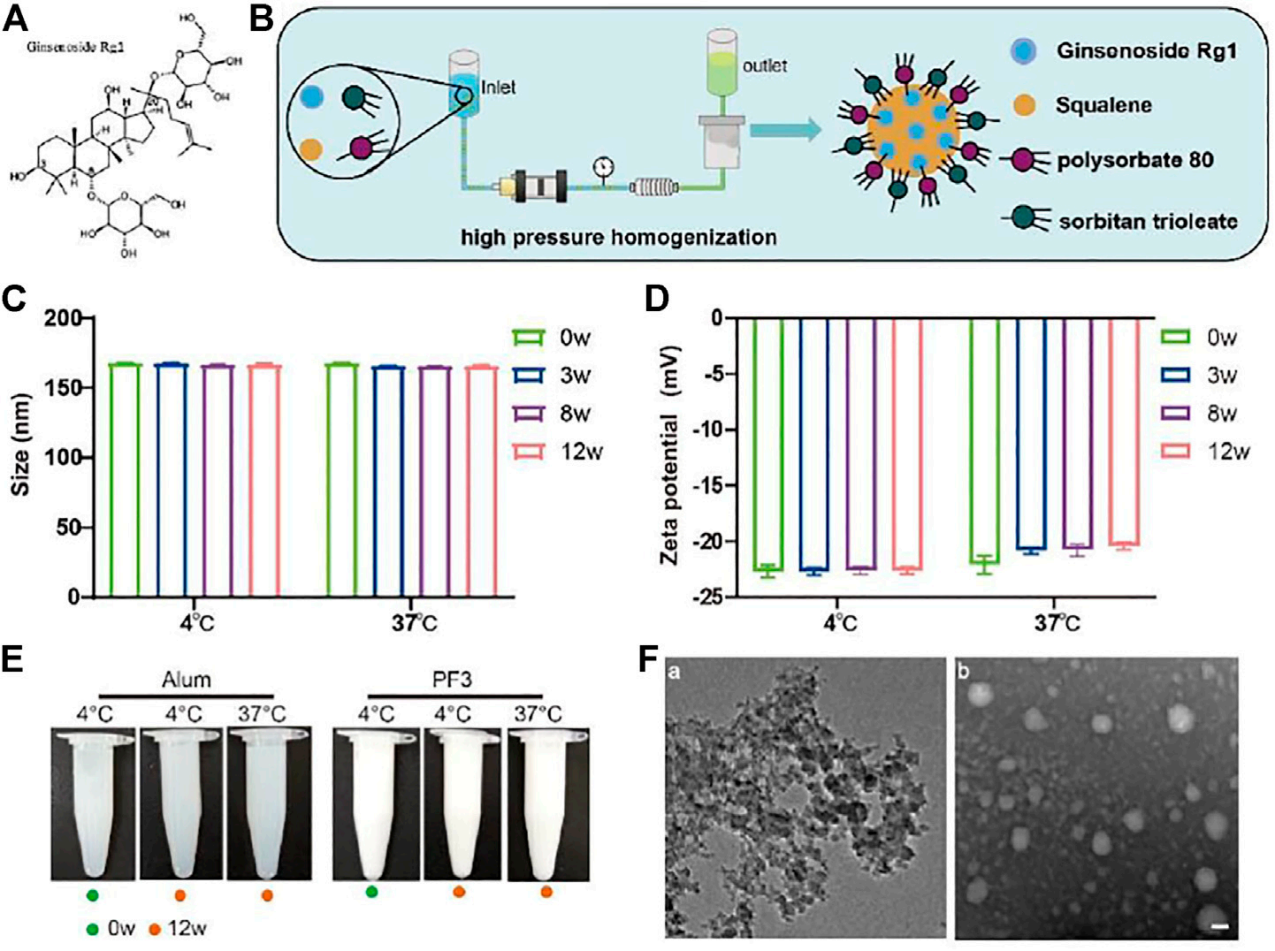
Preparation and characterization of PF3. **(A).** Chemical structure of ginsenoside Rg1. **(B).** Schematic illustration of PF3 preparation process. **(C,D).** Size and Zeta potential of PF3 after 0, 3, 8 and 12 weeks of storge at the indicated temperature. **(E).** Appearance of aluminium hydroxide and PF3 after 0 or 12 weeks of storge at the indicated temperature. **(F).** TEM images of aluminium hydroxide **(a)** and PF3 **(b)**. Scale bar represents 100 nm.

The safety of combined adjuvants is also very important. However, the saponin QS21 in AS01_B_ adjuvant can cause injection-site pain and myalgia (Syed, 2018). Saponins are natural glycosides of steroid or triterpene which exhibited many different biological and pharmacological activities (Sun et al., 2009). Panax ginseng is an edible plant with medicinal effects, such as antitumour, anti-fatigue, immune boosting and anti-aging (Lee and Lau, 2011; Patel and Rauf, 2017). Ginsenoside is an important component of Panax ginseng, and it is a type of immunomodulator that has a wide range of pharmacological activities and significant effects on cardiovascular, central nervous system, endocrine and immune systems (Shin et al., 2015; Ni et al., 2016). Ginsenosides are divided into three types according to the chemical structure of their aglycone: 1) ginsenediol saponins, including Ra1, Ra2, Ra3, Rb1, Rb2, Rb3, Rc and Rd; 2) ginsentriol saponins, including Re, Rf, Rg1, Rg2, Rh1 and notoginsenoside; and 3) ginsenoside of oleanolic acid as a glycoside (Li et al., 2019). Rg1 (Figure 1A) is the main component of ginseng, and it belongs to the protopanaxatriol type (PPT), which has antitumour and steroid hormone effects and improves the non-specific immune function of mice via an unclear mechanism of action. Rg1 plus oil significantly upregulated TLR2 expression but decreased the level of NF-κB in splenocytes. Rg1-oil adjuvant may increase the levels of IL-2 and IL-4 by upregulating TLR2, thus enhancing the immune effect of B. bronchiseptica vaccine in rabbits (Chenwen et al., 2021).

Although the traditional hepatitis B vaccines with aluminum adjuvants can induce high titers of anti-HBs, they cannot cause an adequate cellular immune response to effectively protect hepatocytes from hepatitis B virus (HBV) invasion (Xu et al., 2015). The immune response initiated by the T-cell response to viral antigens is thought to be fundamental for viral clearance and disease pathogenesis in HBV infection (Jung and Pape, 2002). With the development of nanotechnology, nano adjuvants have more advantages, such as enhancing the ability of helping more antigens to enter lysosomes of innate immune cells, or achieving step-by-step multiple stimulus-responsive drug delivery which is similar to that of nanoparticle development in tumor treatment (Yi et al., 2022). In order to overcome weak cellular immunity of aluminum adjuvant, a new nano adjuvant (PF3) was constructed by dynamic high-pressure microfluidization in the present study (Figure 1B). The PF3 is stored at 4°C in solution form as a kind of oil-in-water nano adjuvant. The PF3 consists of ginsenoside Rg1, a natural plant-based immune stimulant, and the MF59-like NE, a nano emulsion which has been marketed and used safely for many years (O'Hagan et al., 2012; O'Hagan et al., 2013; Orsi et al., 2013; Schaffner et al., 2018). Similar to aluminum adjuvant, MF59 induced weak cellular immunity (Del Giudice et al., 2018). The prepared nano adjuvant provided a more comprehensive and balanced humoral and cellular immunologic response and an expected better choice of compound adjuvant for hepatitis B virus than traditional aluminum adjuvant. Moreover, PF3 displayed synergy with Hepatitis B surface antigen (HBsAg) which enhanced cell-mediated immune response. Additionally, PF3 performed excellent biocompatibility *in vivo* with well-controlled immunogenicity and it has good accessibility, low cost and high purity. We anticipate that the new nano PF3 may serve as a safe, accessible and efficient adjuvant.

## Materials and Methods

### Adjuvants Preparation, Antigen, Reagents and Sample for Immunization

Aluminium hydroxide was purchased from Beijing Institute of Biological Products Co., Ltd. (Beijing, China). PF3 (3.9% squalene, 0.47% polysorbate 80 and 0.47% sorbitan trioleate, 0.01% Rg1) was prepared through dynamic high-pressure microfluidization (Microfludics, USA) with the pressure of 10000psi by 7 cycles. Specifically, the oil phase containing squalene and sorbitan trioleate and the water phase containing polysorbate 80 and Rg1 were prepared respectively, then the two were mixed into coarse emulsion, and then the PF3 was prepared through dynamic high-pressure microfluidization. Each batch of PF3 can be up to 20 L in production scale. Squalene, polysorbate 80 and sorbitan trioleate were purchased from Nanjing Weir Pharmaceutical Co., Ltd. (Nanjing, China) and they are all medicinal grade. Ginsenoside Rg1 (purity 99.44%, HPLC) was purchased from Chengdu MUST Bio-Technology Co., Ltd. (Chengdu, China).

Hepatitis B surface antigen (HBsAg) was purchased from the Beijing Institute of Biological Products Co., Ltd. (Beijing, China). HRP Conjugated Goat anti-Mouse IgG1 and IgG2a were purchased from Bethyl Laboratories, Inc. (Montgomery, TX, USA). Anti-HBs kit was purchased from Beijing WANTAI Biotech CO., Ltd. (Beijing, China). Flow cytometry kit was purchased from BD (BD, USA). All other chemical reagents are products of analytically pure grade.

For the preparation of samples for immunization, PF3 (100 μg/ml target concentration of Rg1) was mixed 1:1 v/v with HBsAg antigen (5 μg/ml target concentration).

### Physiochemical Characteristics and Stability of PF3

The particle size, PdI and Zeta potential of PF3 at different time points (weeks 3, 8, and 12) at 4 and 37°C were determined to delineate the physical characteristics and stability. Particle size, PdI and Zeta potential were determined using the ZetaSizer Nano ZS90 dynamic light scattering instrument (Malvern Instruments, United Kingdom). For transmission electron microscopy (TEM,JEOL, Japan) images of PF3 and aluminium hydroxide, first, put a drop of sample (about 30 μl) to the supporting film, stay for about 10min, then put the dried supporting film on the sealing film, put a drop of uranium dioxy acetate dye, stay for 90s, and then dry on the filter paper for 3 h for observation.

### Mice and Method of Euthanasia

Eight-week-old, specific pathogen free (SPF) female BALB/c mice were purchased from Beijing Vital River Laboratory Animal Technology Co., Ltd. (Beijing, China) and randomly divided into several different groups, with starting weights between 16 and 18 g. The experiment personnel are blind to each immunized group. The Animal Ethics Committee of the National Vaccines and Serum Institute (AECNVSI) approved the animal experiments and all procedures. Mice were euthanized via an intraperitoneal injection of 80 mg/kg sodium pentobarbital (Serva, Germany) and blood or spleens were collected.

### Grouping of Mice and Immunization Procedures

The mice were divided into 3 groups of 10 mice: S(HBsAg), S + Al(OH)_3_ and S + PF3. Mice were immunized at weeks 0 and 3 via intramuscular injection, 0.5 μg/100 μl HBsAg per mouse for one dose, and blood was collected from 5 mice per group 3 weeks after immunization for the quantitative detection of anti-HB antibodies and IgG1 and IgG2a antibody subclass detection using enzyme-linked immunosorbent assay (ELISA). One week after booster immunization, the spleens of 5 mice per group were collected for enzyme-linked immunospot assay (ELISpot) and intracellular cytokine staining (ICS) testing using fluorescence-activated cell sorting (FACS).

### Determination of Serum Anti-HBs and IgG1 and IgG2a Antibody Response

Serum anti-HBs and IgG1 and IgG2a antibody levels were determined using indirect ELISA kits. Serum was collected 3 weeks after immunization. Quantitative detection of anti-HBs (mIU/ml) antibodies was performed using commercially available ELISA kits. For IgG1 and IgG2a (ng/ml) antibody determination, ninety-six well plates were coated with 1 μg/ml HBsAg overnight, and the plate was blocked with 2% bovine serum albumin (BSA) for 1 h then washed 5 times with 1‰ Tween 20-PBS. The initial series of diluted mouse serum was added and incubated at 37°C for 1 h. After washing, HRP-labelled sheep anti-mouse IgG1 or IgG2a antibodies (1:10,000 dilution) were added, and incubated at 37°C for 1 h. A chromogenic solution (containing substrate A hydrogen peroxide and substrate B tetramethylbenzidine (TMB)) was added and incubated at 37°C for 10 min and the termination solution was added. The absorbance value at dual wavelengths of 450/630 nm was read. Four Parameter Logistic (4 PL) Regression was used to calculate the IgG1 and IgG2a antibody concentration (ng/ml).

### Determination of Cell-Mediated Immune Response

Specific T-cell responses to HBsAg were measured using ELISpot and ICS. One week after the booster immunization, mice were sacrificed. Splenocytes were isolated from the spleen and stimulated with a specific S peptide (S28-39, *IPQSLDSWWTSL*, H-2d restricted). Briefly, for ELISpot, mice spleen cells were isolated and added into each well with 50,000 cells and 1 μg stimulating peptide per well. ConA was used as positive control. After incubation at 37°C for 20 h, biotin-labeled antibody (Mabtech, Sweden; IFN-r or IL-2) was added, followed by substrate coloration, water washing terminated, and plate reading counting after drying. For ICS, 250 μl of flow fluid (2% FBS PBS) was added to each well of the 96-well cell plate, and centrifugation was performed at 200 g for 5 min. Then, 50 μl of FITC-CD8 (BD, USA) and PerCP-CD3 (BD, USA) dyes were added to each well for surface dyeing. After centrifugation at 200 *g* for 5 min, 250 μl of flow fluid was added to each well, and 100 μl Fixation/Permeabilization solution (BD, USA) was added each well. The reaction was performed at 4°C for 20 min in the dark. Then, 50 μl of PE-IFN-γ and APC-IL-2 dyes (BD, USA) diluted in Perm/Wash buffer (BD, USA) were added to each well. After washing, 200 μl of flow solution was added to each well, and the results were detected using flow cytometry (BD FACS Canto™ II, USA). PMT voltages are as follows, P2 385V, P3 607V, P4 565V, P5 557V, P7 668V. Data are presented as the means ± SD and are representative of one of two independent experiments with similar results.

### Safety

The safety of the new nano adjuvant PF3 was evaluated using changes in body weight and histopathological changes of heart, liver, spleen, lung and kidney by HE staining of sections 7 days after injection. Meanwhile, histopathological changes at the intramuscular injection site were observed at 4 and 72 h. The injection dose for safety was the same as the immunoassay.

### Statistical Analysis

GraphPad Prism 8.0 software (GraphPad Software Inc., San Diego, CA, USA) was used for data analyses and statistical tests. The data were measured with mean standard deviation. Means were compared using Student-t test and one-way analysis of variance (ANOVA) followed by a Tukey-Kramer post hoc test using a 95% confidence interval. *p* values of less than 0.05^∗^, 0.01^∗∗^, 0.001^∗∗∗^ and 0.0001^∗∗∗∗^ were considered as statistically significant.

## Results

### PF3 has Stable Physiochemical Characteristics

In order to evaluate the physicochemical stability of PF3, particle size and Zeta potential were measured at 4 and 37°C, the latter is a destructive condition for acceleration. The tolerance and stability of PF3 formulation were investigated to provide data supporting for long-term storage at 4°C. The particle size of PF3 was 168.1 nm and the Zeta potential was -22.8mV at 4°C. The results of particle size and Zeta potential at 37°C at different time (3, 8 and 12 weeks) showed that PF3 displayed relatively stable physicochemical characteristics (Figures 1C,D). There was no obvious change in appearance of aluminium hydroxide and PF3 after 0 or 12 weeks of storge at 4°C or 37°C. These results suggest that PF3 may be stored stably at 4°C for a much longer time. The results of TEM showed that PF3 presented uniform nanoparticles, while the aluminum hydroxide presented irregular particles of uneven size (Figure 1E), which is similar to the DLS results (particle size 168.1 ± 2.483nm, PdI 0.128 ± 0.007 for PF3; particle size 12,980 ± 289.9nm, PdI 0.395 ± 0.006 for aluminum hydroxide).

### PF3 Enhanced the Antibody Responses to HBsAg

**FIGURE 2:**
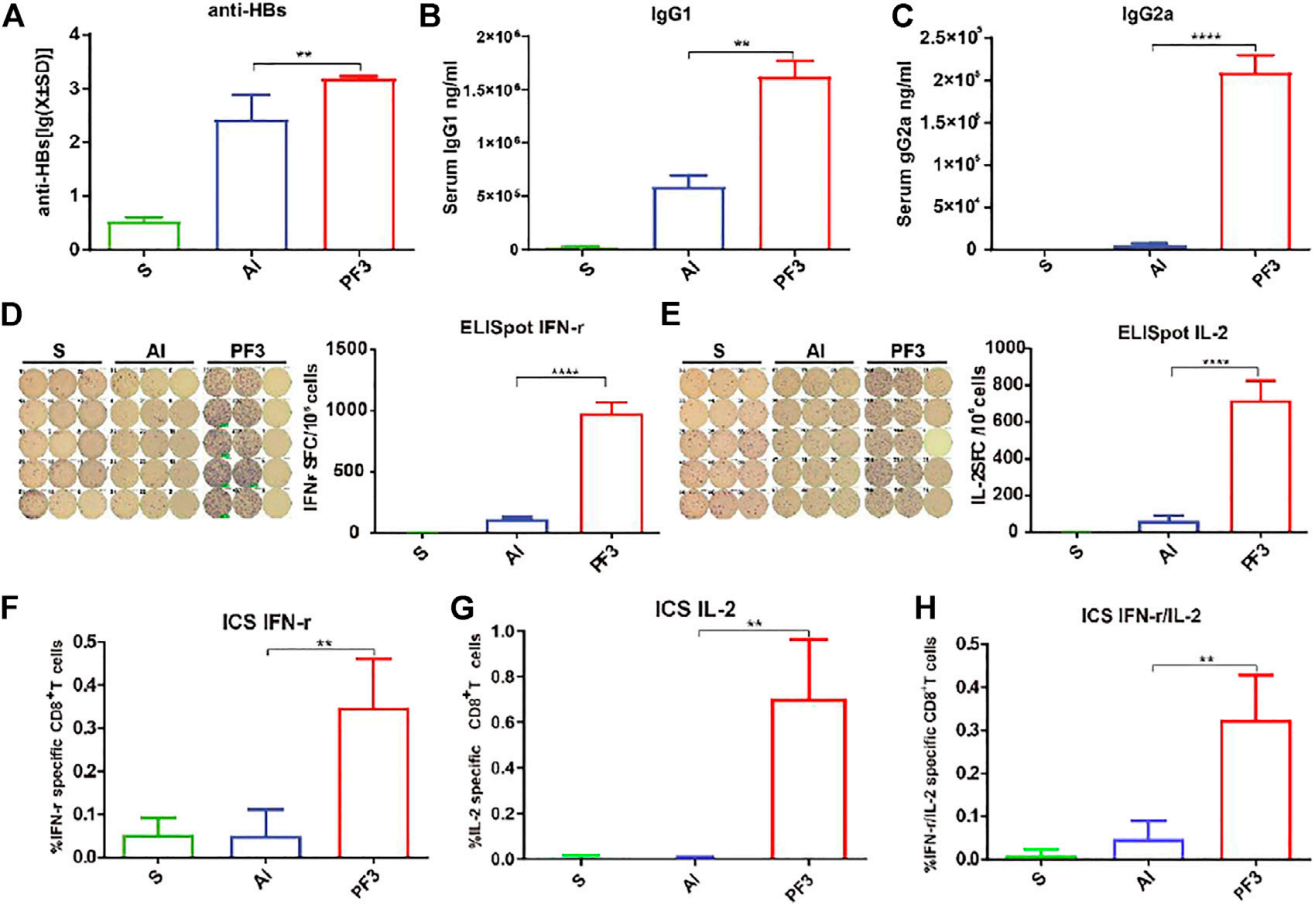
Serum anti-HBs and IgG1 and IgG2a antibody levels of mice and specific T-cell responses measured using ELISpot and ICS immunized with HBsAg combined with different adjuvants. BALB/c mice (n = 5) were immunized intramuscularly with HBsAg (5 μg/ml) combined with Al (500 μg/ml), PF3(50 μg/ml) or Rg1 (50 μg/ml). **(A).** anti-HBs (mIU/ml); **(B,C).** IgG1 and IgG2a (ng/ml); **(D,E).** ELISpot IFN-r and IL-2; **(F–H).** ICS IFN-r and IL-2. Data are presented as the means ± SD and are representative of one of two independent experiments with similar results. Note: ∗*p* < 0.05, ∗∗*p* < 0.01, ∗∗∗*p* < 0.001 and ∗∗∗∗*p* < 0.0001.

It is well known that the aluminum adjuvant has a strong humoral immunity enhancing effect, and the anti-HBs antibodies play an important role in clearing viral infection. For the immunopotentiation of the PF3 adjuvant to HBsAg, the humoural immunity results 3 weeks after 1 dose showed that the PF3 had a significantly higher mean serum antibody level than aluminium hydroxide (1552.74 vs. 268.35, 5.79 times;*p* < 0.01; Figure 2A). IgG1 and IgG2a level in serum showed similar results, the serum IgG1 (1623923.00 vs. 587,830.25, 2.76 times) and IgG2a (209,790.85 vs. 5344.41, 39.25 times) level of the PF3 was significantly higher than aluminium hydroxide (*p* < 0.01 and *p* < 0.0001, respectively; Figures 2B,C). Therefore, PF3 showed a higher humoral immune-enhancing effect than aluminium hydroxide for the hepatitis B virus, and the higher IgG2a levels suggested that the Rg1 in the PF3 may play an enhanced cellular immunity role as a stimulus molecule for HBsAg to a much higher extent (Figure 2C).

### PF3 Enhanced the Cell-Mediated Immune Response to HBsAg

Although aluminum adjuvant has a strong humoral immunity enhancing effect, it has a weak cellular immunity effect. To assess the ability of PF3 to elicit a cellular immune response when administered intramuscularly with HBsAg, PF3-induced IL-2 and IFN-γ (as markers of the Th1 response) secreting cells in the spleen were detected using ELISpot and ICS. As shown in Figures 2D,E, mice vaccinated with PF3 produced significantly higher counts of specific IFN-γ and IL-2-expressing cells (SFCs, Spot Forming Cells) than aluminium hydroxide (978.50 vs. 113.75, 8.60 times for IFN-γ; 715.50 vs. 62.00, 11.54 times for IL-2; *p* < 0.0001 and *p* < 0.0001, respectively). The results of ICS were similar to ELISpot. IFN-γ, IL-2 and IFN-γ/IL-2 expression in CD8^+^ T cells of the PF3 group showed a significantly higher enhancement than the aluminium hydroxide (0.35 vs. 0.05%, 6.87 times for IFN-γ; 0.71 vs. 0.01%, 94.00 times for IL-2; 0.33 vs. 0.05%, 6.89 times for IFN-γ/IL-2; *p* < 0.01, *p* < 0.01 and *p* < 0.01, respectively. Figures 2F–H). These results suggest that PF3 can significantly improve the cellular immunity against HBsAg compared with aluminum adjuvant.

### PF3 is Safe in Mice

**FIGURE 3:**
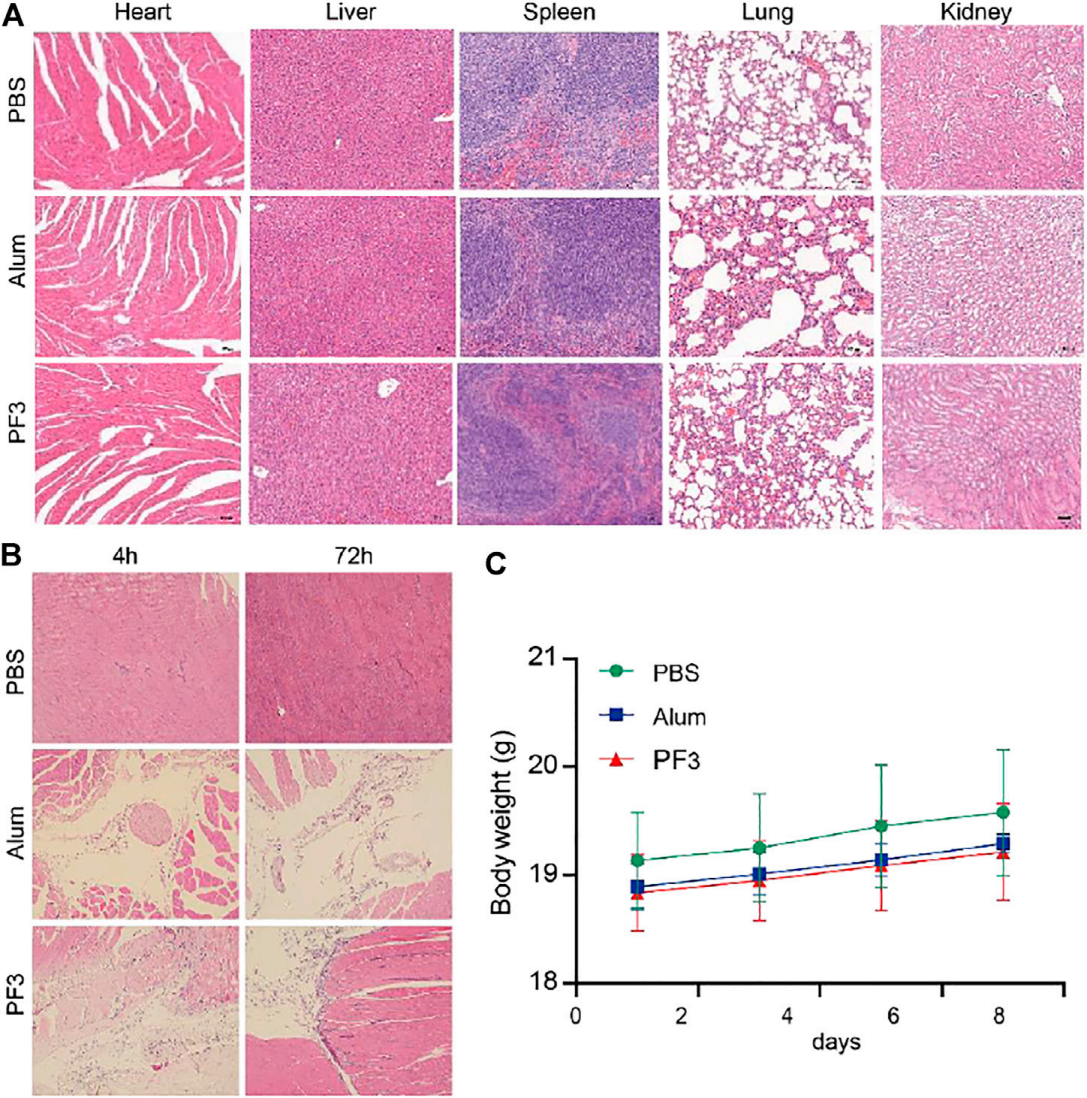
PF3 is Safe in Mice. **(A)** Representative images of biocompatibility evaluations via H&E staining of vital organ sections. Scale bar represents 100 µm. **(B)** HE staining of muscle at different time after intramuscular injection. **(C)** Change of the body weight of BALB/c mice (n = 3).

Changes in morphology and inflammatory cell infiltration of the major organs including the heart, liver, spleen, lungs, and kidneys were observed 7 days after injection of the PBS, Alum or PF3. The histopathological results showed that PBS group were normal and almost no inflammatory cell infiltration 72 h after intramuscular injection. No evident pathology was found in the major organs and no significant difference was observed between the PF3 group and the aluminum hydroxide group (Figure 3A). No significant differences were observed of histopathological change at the intramuscular injection site at 4 and 72 h between the PF3 group and the aluminum hydroxide group (Figure 3B). As indicated in Figure 3C, the body weight of mice showed a steady increase trend among the three groups, and no significant difference was observed. Therefore, PF3 exhibited well-controlled immunogenicity and acceptable biosafety as a kind of HBV vaccine adjuvant candidate.

## Discussion

Hepatitis B is a kind of infectious liver disease caused by hepatitis B virus, mainly including acute hepatitis and chronic hepatitis. While HBV may also cause cirrhosis and primary liver cancer. Hepatitis B has become a major global health problem, infecting nearly 350 million people worldwide and killing about 60,000 of them each year (Rein et al., 2012). Hepatocellular carcinoma (HCC) accounts for 70–90% of primary hepatic carcinoma cases (Yu and Yuan, 2004). It is the third leading cause of cancer-related deaths worldwide and is expected to get worse in the coming years (Venook et al., 2010). So far, hepatitis B Vaccine (HepB) is the most effective way to prevent HBV infection and transmission, with an effective rate of 85–95%. However, there are still 5–10% of the population with no response or low response. The risk groups for poor response include those with immunosuppression or dialysis-dependent or end-stage renal disease. Therefore, immunogenicity enhanced vaccines are needed to protect them from hepatitis B infection (Saco et al., 2018). In addition, the level of anti-HBs decreased with the increase of time after vaccination, with the fastest decline in the first year after immunization and gradually decreased thereafter. The persistence of antibody was positively correlated with the peak response after the initial vaccination (Honorati et al., 1999). Cellular immunity is also important for hepatitis B virus clearance, and one reason for the weak cellular immunity of aluminum adjuvants is that antigens are processed through the lysosomal pathway and presented via major histocompatibility complex II (MHC-II), instead of cross-presentation for MHC-I-mediated cellular immunity (Peng et al., 2020). To achieve the complete clearance of HBV, it is vital to augment strong cellular immunity and cytotoxic T lymphocytes (CTLs) (Hu et al., 2020).

Although there are a variety of new adjuvants developed, they also have some inevitable shortcomings, such as the need to improve the stability of liposomes, some adjuvants have strong adverse reactions, some are poor in water solubility, and some cost too much and are complicated to prepare.

The present study showed that PF3 induced stronger humoral immunity and cellular immune response than aluminium hydroxide when combined with the HBsAg antigen. Besides, it induced higher IgG2a, IFN-γ and IL-2 levels, which means that it may induce a more balanced Th1/Th2 response that compensates for the weak Th1-type response induced by aluminium hydroxide. Moreover, PF3 also showed a strong cellular immune enhancement on VZV (varicella zoster virus) recombinant gE antigen, demonstrated by the method ELISpot and flow cytometry ICS (data not shown), which suggest that PF3 can be a potential candidate adjuvant for recombinant herpes zoster vaccine. In addition, PF3 had no obvious inflammation in the major organs, exhibiting comparable safety to aluminium hydroxide. In terms of ginsenosides, they have strong efficacy and good safety in antitumour and other aspects (Huang et al., 2017). Moreover, it is easy to extract and purify with very high purity and lower costs for Rg1. In terms of safety, the PF3 infiltration of inflammatory cells at the intramuscular injection site recovered better at 72 h than at 4 h compared with aluminum hydroxide (Figure 3B). These results suggest that PF3 have a faster inflammatory recovery at the site of intramuscular injection than aluminum hydroxide.

In conclusion, our data demonstrated that the new nano adjuvant PF3 produced a range of immunological adjuvant effects in HBsAg immunized mice and better humoral and cellular immunity than aluminum adjuvant. PF3 also showed favourable safety characteristics and acceptable stability. Because of easy preparation, low cost, simple dosage form and water soluble, PF3 could be combined with other antigen to construct novel vaccines to induce strong immune response.

## Data Availability

The original contributions presented in the study are included in the article/Supplementary Material, further inquiries can be directed to the corresponding author.
