# Supplementary figures and images for: A New Nano Adjuvant of PF3 Used for an Enhanced Hepatitis B Vaccine
Source: Front Bioeng Biotechnol. 2022 May 10;10:903424. doi: 10.3389/fbioe.2022.903424 (PMC9127465; doi:10.3389/fbioe.2022.903424)

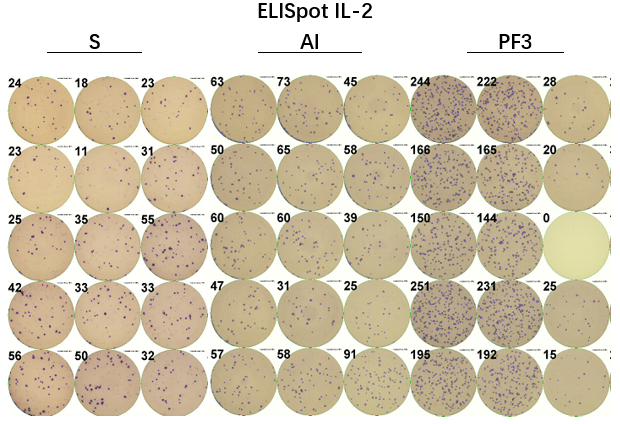

Supplement: Supplementary file 1 [file Image2.PNG]

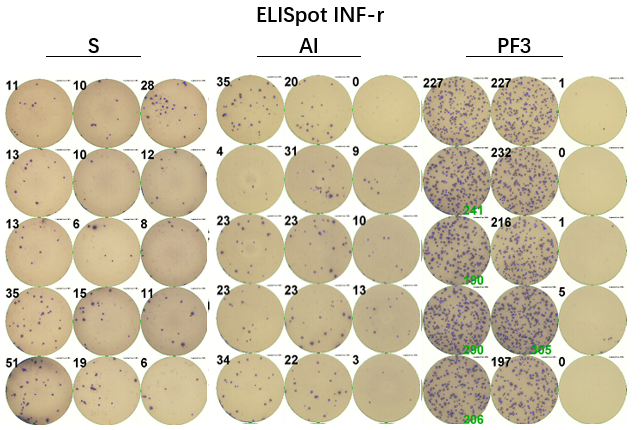

Supplement: Supplementary file 2 [file Image1.PNG]

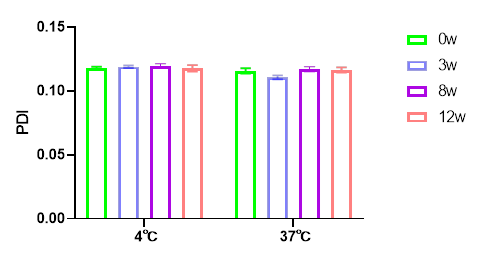

Supplement: Supplementary file 3 [file Image3.PNG]
